# Radiological assessment of pulmonary vascularity in congenital heart disease: standardized clinician assessment versus deep learning–based prediction

**DOI:** 10.1186/s44348-026-00084-7

**Published:** 2026-08-21

**Authors:** Nisanth Selvam, Dilip Parasu, Akshay Desale, Pranoti Toshniwal, Taniya Rachel Issac, Shine Kumar, Navaneetha Sasikumar, Shilpa Radhakrishnan, Raman Krishna Kumar

**Affiliations:** 1 Department of Paediatric Cardiology, Amrita Institute of Medical Sciences, Kochi, India; 2 Amrita School of Artificial Intelligence, Amrita Viswa Vidyapeetham, Coimbatore, India; 3 Department of Radiology, Manipal Hospital, Pune, India; 4 Department of Paediatric Cardiology, Sri H.N. Reliance Foundation Hospital and Research Centre, Navi Mumbai, India; 5 Department of Cardiology, University of Alberta, Stollery Children’s Hospital, Edmonton, AB Canada; 6 Department of Radiology, Amrita Institute of Medical Sciences, Kochi, India

**Keywords:** Pulmonary circulation, Chest Xray, Congenital heart defects, Artificial Intelligence

## Abstract

**Background:**

Assessment of pulmonary vascularity on chest radiographs (CXRs) in congenital heart disease (CHD) is limited by subjectivity, and existing criteria lack sufficient validation. Artificial intelligence-based deep learning model (DLM) analysis may improve accuracy. We developed and validated structured criteria and a DLM to evaluate pulmonary vascularity and compared it with conventional clinical interpretation.

**Methods:**

A total of 400 deidentified CXRs from CHD patients with Fick-derived pulmonary-to-systemic flow ratio (Qp:Qs) were evaluated by three experts and three trainees in pediatric cardiology and radiology using nine prespecified criteria. A separate set of 1,088 CXRs was used to train the DLM. The clinician-interpreted CXRs were subsequently assessed by the DLM to predict the Qp:Qs.

**Results:**

Intraclass correlation between Fick- and DLM-derived Qp:Qs was 0.782 (*P* < 0.001), with a root mean squared error of 0.395 (*P* < 0.001). The DLM showed a strong agreement with Fick-derived classification of pulmonary vascularity (κ = 0.770, *P* < 0.001; predicting Qp:Qs > 1.5: 90% specificity, 98% sensitivity, area under the receiver operating characteristic curve [AUROC] 98%; predicting Qp:Qs < 0.9: 92% specificity, 95% sensitivity, AUROC 98%). For interpreting pulmonary vascularity as increased or decreased, clinician concordance was 75% and 66%, respectively, whereas DLM concordance was 88% and 98%, respectively (*P* = 0.03). Among the structured criteria, the number of end-on vessels demonstrated strong interobserver agreement, whereas agreement for individual peripheral vessels, the size of end-on vessels, branch pulmonary arteries with their respective bronchi, and opacity of lung fields was modest.

**Conclusion:**

Deep learning–based analysis of CXRs enables accurate evaluation of pulmonary vascularity and outperforms structured assessment by clinicians.

**Graphical Abstract:**

Central illustration

**Figure:**
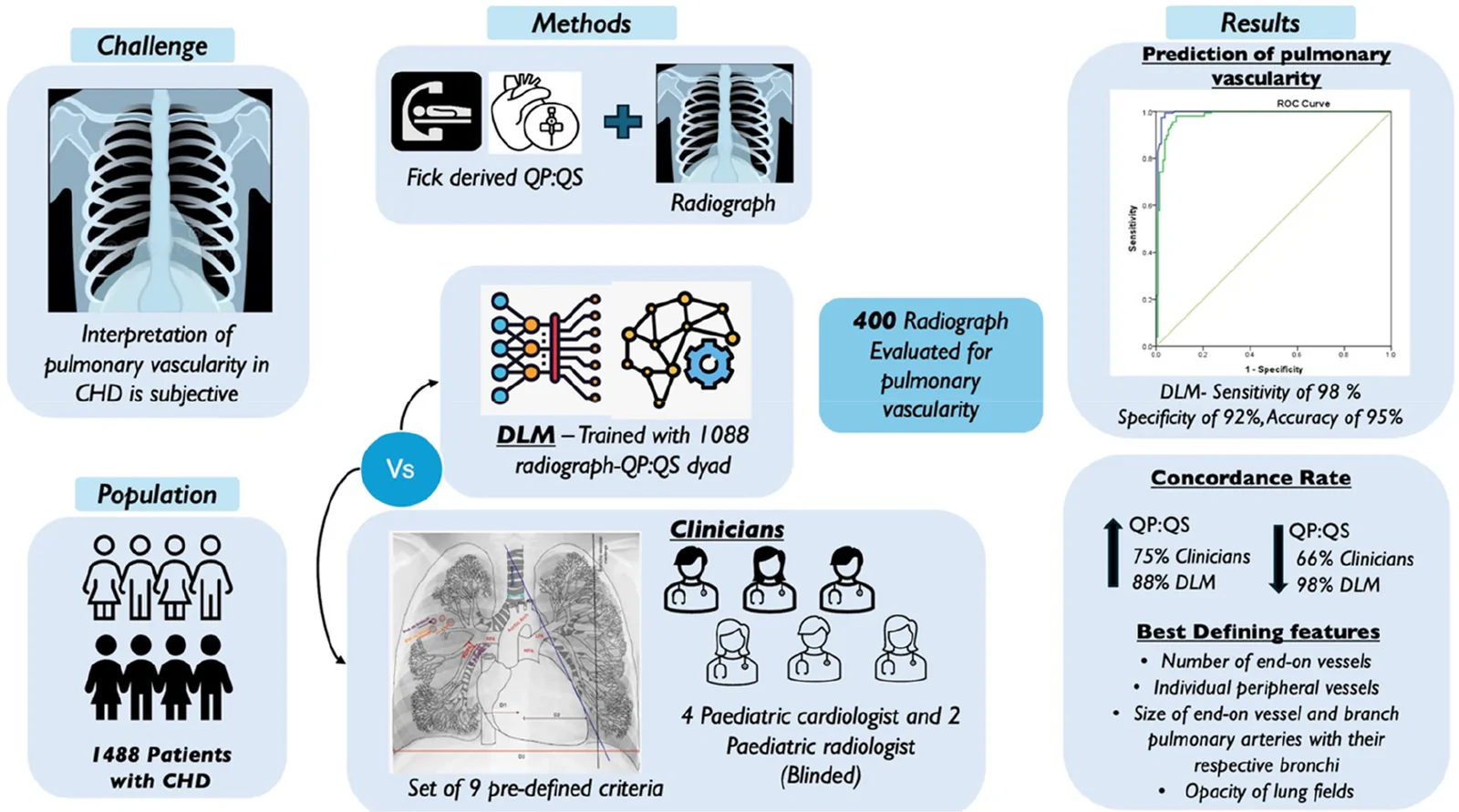

## Background

The relative flows and resistance across the systemic and pulmonary circuits dictate the overall physiology, clinical presentations, and management of many congenital heart diseases (CHD) [1–3]. Therefore, quantification of pulmonary-to-systemic blood flow ratio (Qp:Qs) and pulmonary and systemic vascular resistances are often an integral component in the management of CHD.

Although several modalities can estimate Qp:Qs, an accurate assessment generally requires either an invasive cardiac catheterization or cardiac MRI [4–7]. Chest radiograph (CXR) is traditionally considered useful in visualizing lung vasculature. However, assessment is often subjective with inconsistent accuracy. In addition, the criteria for assessment of pulmonary vascularity in CHD on CXRs are not standardized and not validated [8–12].

Recently, artificial intelligence (AI)-based deep learning models (DLMs) have been successfully applied in the detection of lung cancer, tuberculosis, pneumonia, pulmonary artery hypertension, and pulmonary oedema [13–18]. There is currently limited data on the application of DLMs for assessment of pulmonary vascularity on CXR in CHD [19], and this has not been compared with standardized assessment by clinicians and radiologists.

Therefore, the objectives of this study were to determine the accuracy and interobserver agreement of visual assessment of pulmonary vascularity among clinicians and radiologists using a structured protocol and to develop and validate a DLM for quantitative assessment of pulmonary vascularity in predicting shunt ratio and compare its performance with structured visual assessment.

## Methods

### Ethics statement

The study was approved by the Institutional Ethics Committee of Amrita Institute of Medical Sciences (No. ECASM-AIMS-2023–555). The waiver for informed consent was granted as the radiographs were anonymized.

### Study design and setting

This prospective study was conducted in the Department of Paediatric Cardiology at a tertiary referral center in South India and an academic center for AI between October 2023 and April 2025.

The inclusion criteria were stable patients with CHD who had undergone cardiac catheterization with a documented Qp:Qs using the direct Fick method and who had a CXR obtained within 1 week of cardiac catheterization at rest, breathing room air. CXR quality was confirmed by appropriate positioning, absence of rotation, adequate exposure, and sufficient lung expansion. CXRs were retrieved from our digital radiography system (Digital Diagnost 4 High Performance, Philips Healthcare). Patients requiring any form of ventilatory or oxygen support, those with hemodynamic instability or nondocumentation of Qp:Qs in the cardiac catheterization report, or suboptimal CXR quality and differential vascularity were excluded from the study.

We collected baseline demographic data, clinical details, CXRs of appropriate quality, and Qp:Qs. The Qp:Qs was classified into three categories: decreased (< 0.9), normal (≥ 0.9 to < 1.5), and increased (≥ 1.5). Nine predefined criteria were identified for classifying pulmonary vascularity as normal, increased, or decreased in CXR (Table 1) [20–22].

**Table 1:** Information sheet describing individual defining features

No.	Feature	Classification	Image
1	RDPA and tracheal diameter RDPA is measured at the level where it parallels the right main bronchus Tracheal diameter is measured just above the impression of the aortic arch	Increased vascularity: difference >2 mm Normal or decreased vascularity: equal or smaller RDPA	
2	Branch PA size and adjacent bronchi Normally, the main branch PA are 1.3–1.4 times the size of their adjacent bronchi	Any discrepancy is abnormal Above 1.5 times is considered increased	
3	Prominence of the MPA Normally, when a tangential line is drawn from the aortic knob to the left edge of the heart, the pulmonary artery should lie medial to that line	Decreased vascularity: empty pulmonary bay Increased vascularity (may also be seen in pulmonary hypertension): MPA knuckle lateral to the line	
4	Individual peripheral vessels In a normal radiograph, peripheral vessels gradually taper from the hilum to the periphery	Increased pulmonary vascularity: vessels are dilated or sometimes tortuous Decreased vascularity: vessels are not clearly visualized	
5	Bronchovascular marking Normal markings extend to two-thirds of the lung field	Increased vascularity extends up to the lateral one-third Decreased vascularity is limited to the hilar region	
6	No. of end-on vessels	More than 3 in the unilateral lung field or >5 in both lung fields combined is considered a feature of increased pulmonary vascularity End-on vessels are small and almost not identifiable in decreased vascularity	
7.	Size of end-on vessel and end-on bronchus Usually, end-on vessels are the same size as end-on bronchi	Increased vascularity is observed when end-on vessels are at least 1.5 times the size of end-on bronchi	
8.	Opacities of lung fields	Increased opacities in the lung fields and obscuration of the margins of the vessels are suggestive of increased vascularity A hyperlucent lung with no opacities is seen in decreased vascularity	
9.	Heart size/individual chamber dilatation Cardiomegaly is defined as increased CTR >0.6 in infancy, 0.55 up to 5 years, and 0.5 in older children and adults	Cardiomegaly is more suggestive of increased vascularity Normal CTR can be a feature of decreased vascularity But there are always exceptions to this	

*CTR* cardiothoracic ratio, *MPA* main pulmonary artery, *PA* pulmonary artery, *RDPA* right descending pulmonary artery

**Fig. 1:**
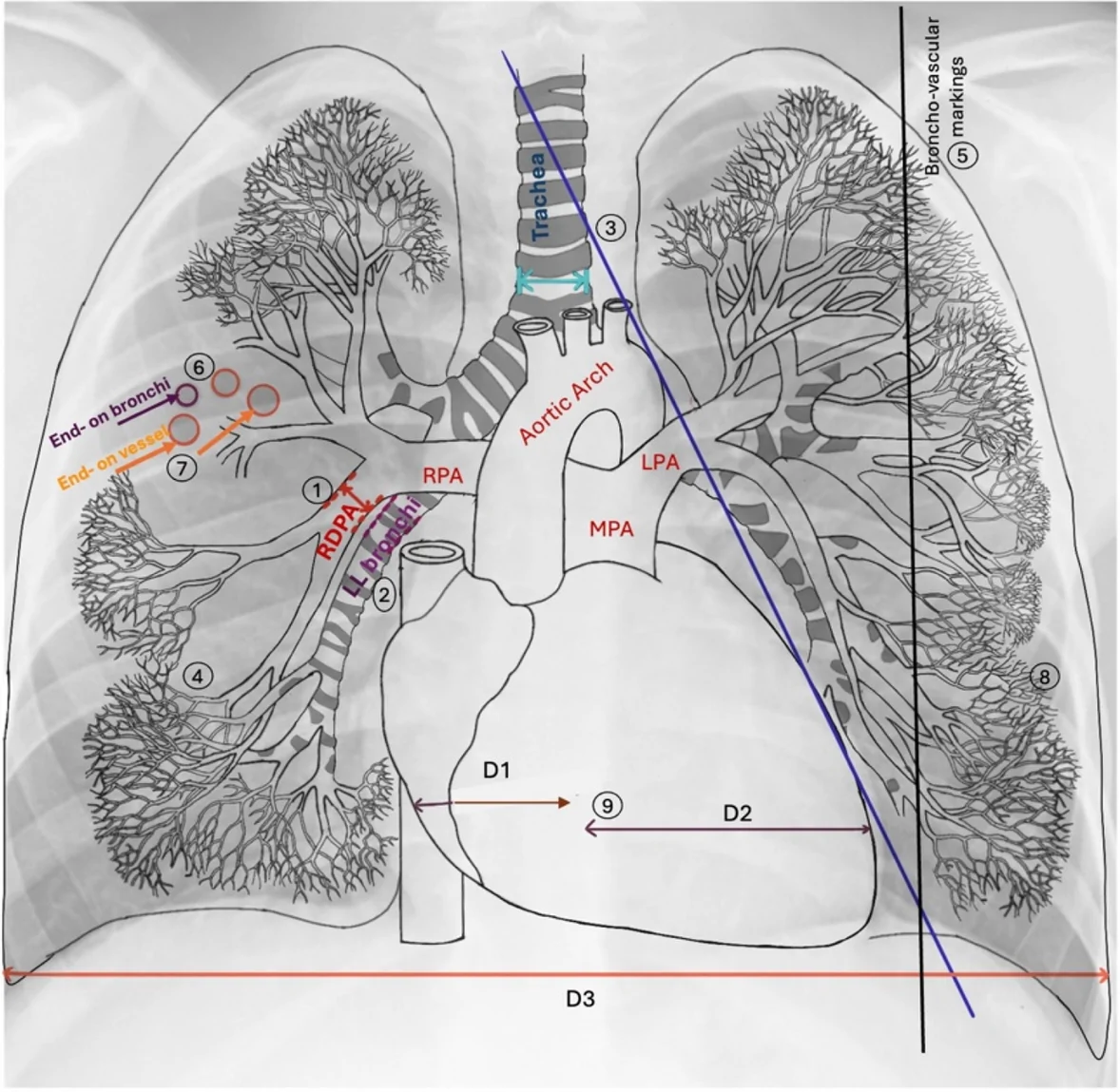
Pictorial representation of the defining features of pulmonary vascularity on chest radiograph. (1) Right descending pulmonary artery (RDPA) and tracheal diameter. (2) RDPA and right lower lobe bronchi. (3) Tangential line from aortic knuckle to left heart border. (4) Individual peripheral vessels. (5) Bronchovascular marking. (6) Number of end-on vessels. (7) Size of individual end-on vessel and respective end-on bronchi. (8) Opacity of lung fields. (9) Cardiothoracic ratio (D1 + D2/D3). LPA, left pulmonary artery; MPA, main pulmonary artery; RPA, right pulmonary artery

A sample size of 400 was derived from previous literature on the sensitivity of CXRs in the assessment of pulmonary vascularity [11, 19]. A designated set of 400 anonymized digital CXRs was interpreted using predefined criteria (Fig. 1) by three senior staff members (two pediatric cardiologists and one pediatric radiologist) and three trainees (two pediatric cardiologists and one pediatric cardiac radiologist).

In parallel, the DLM was developed and trained using 1,088 CXRs and Qp:Qs values (training group) from our database. The radiographs were downloaded from the hospital database in DICOM format and manually converted to PNG format. Before being used for model training, they were manually trimmed to remove extrathoracic structures, retaining only the thoracic cage from the apex of the lung to the diaphragm. The 400 CXRs interpreted by the experts and trainees were subsequently evaluated using the DLM to determine pulmonary vascularity (evaluation group).

### Deep learning model

We used transfer learning in our study, leveraging a pretrained DLM as a source task and adapting it to the new task. This approach reduces the learning cost and improves accuracy with limited datasets.

We evaluated multiple model backbones, including DenseNets, Inception V3, ResNet variants (ResNet18, ResNet34), VGG variants, and Vision Transformers (ViT).

ViT models consistently outperformed convolutional neural networks (CNNs) [23]. ViT is a deep learning transformer-based architecture that divides an image into tokens and processes them through the architecture to generate predictions. Models were chosen from the PyTorch Image Models (timm) repository, which provides pretrained ImageNet weights (https://www.image-net.org). Among the models, the ViT 16 base model was found to outperform the others.

The model was adjusted to input layers for grayscale images and modified output layers from classification to a regression layer to predict continuous Qp:Qs values. The ViT-Base-16 model that achieved the best results, with superior capability to capture the overall image context and predict Qp:Qs, was used in this study.

CXR images were organized systematically into folders. A Microsoft Excel sheet (Microsoft Corp) was created to map each image to its respective Qp:Qs value.

**Fig. 2:**
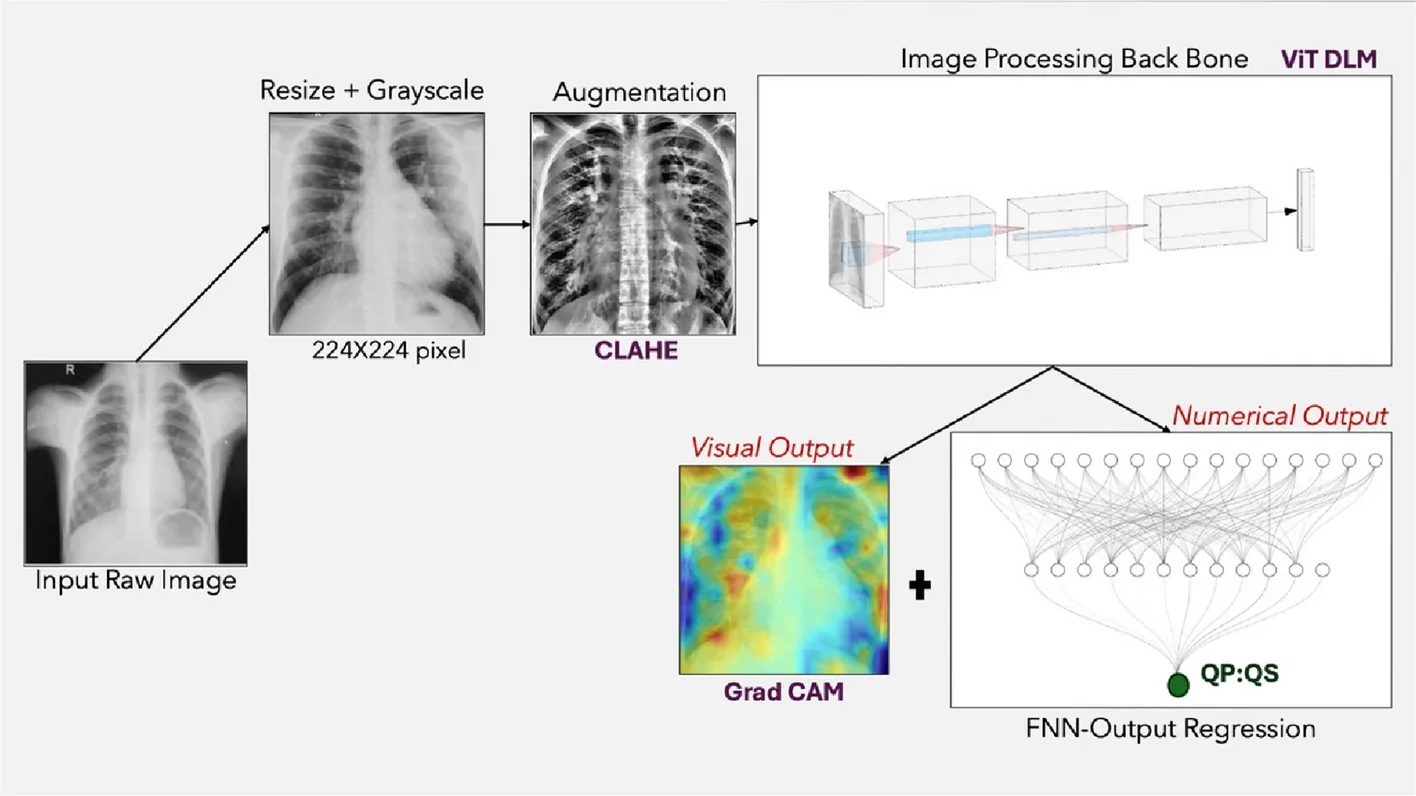
Simplified pictorial representation of the deep learning model’s data execution and output. CLAHE, contrast-limited adaptive histogram equalization; DLM, deep learning model; FNN, fully connected neural network; Qp:Qs, pulmonary-to-systemic blood flow ratio; ViT, Vision Transformer

Images were resized to the input dimensions required by the models (224 × 224 pixels). As radiographs are inherently grayscale, the model was adapted to handle single-channel inputs. Augmentation techniques applied included random horizontal flip rotations (up to 20°) and contrast-limited adaptive histogram equalization (CLAHE) [24]. Both CLAHE and augmentation effects were evaluated to understand their impact on model performance. The model used root mean squared error (RMSE) for evaluation, as it provides a direct measure of the prediction error in the same units as the output quantity [25]. The Adam optimizer (with varying learning rates), a batch size of 16, and training over 50 epochs were set as hyperparameters. Figure 2 summarizes the application of the DLM for radiograph assessment.

### Statistical analysis

Categorical variables were expressed as percentages, and continuous variables as median with interquartile range (IQR) for skewed distribution and mean with standard deviation (SD) for normal distribution. A scatter plot was used to depict the relationship between the Fick- and DLM-predicted Qp:Qs. Agreement between the Fick- and DLM-derived Qp:Qs was assessed using Bland–Altman analysis, intraclass correlation, and RMSE. Agreement between classification based on Fick method and DLM-predicted Qp:Qs was evaluated using Cohen κ. Diagnostic concordance between classification based on Fick-derived Qp:Qs and the classification by clinicians was evaluated using the McNemar test. The sensitivity and specificity of the DLM-based assessment of Qp:Qs was determined using area under the receiver operating characteristic curve (AUROC). The interobserver agreement between experts and fellows was assessed using Cohen κ. The interobserver agreement on the predefined features of pulmonary vascularity was determined using Kendall W. The statistical analysis was done using IBM SPSS ver. 25.0 (IBM Corp). A two-tailed *P*-value of < 0.05 was considered statistically significant.

## Results

### Demographics

A total of 1,488 patients were included in the study. Of these, the DLM training group included 1,088 patients, with a median age of 8 years (IQR, 4–21 years) and female predominance (54.4%). Most patients had acyanotic CHD (58.3%), predominantly shunt lesions (56.4%). The median Fick-derived Qp:Qs was 1.40 (IQR, 0.69–2.17). Overall, 363 (33.4%) had Fick-derived Qp:Qs < 0.9, 514 (47.2%) had Qp:Qs > 1.5, and the remainder had a Qp:Qs between 0.9 and1.5. The 400 patients in the evaluation group had similar baseline characteristics to the training group (Table 2). The median Fick-derived Qp:Qs was 1.50 (IQR, 0.72–2.20); 194 (48.5%) had a Fick-derived Qp:Qs > 1.5, 118 (29.5%) had a Qp:Qs < 0.9, and the rest had a Qp:Qs between 0.9 and 1.5. The majority of the CXRs (98.2%) were performed the day before the catheterization.

**Table 2:** Baseline characteristics of the study population (*n* = 1,488)

Characteristic	Training group **(***n* = 1**,**088**)**	Evaluation group **(***n*** = **400**)**
**Age (yr)**	8 (4–21)	8 (4–21)
Height (cm)	123 (102–153)	126 (99.25–155.5)
Weight (kg)	21.5 (14.0–45.0)	22.9 (13.7–46.95)
**Sex**		
Male	496 (45.6)	183 (45.8)
Female	592 (54.4)	217 (54.2)
**SpO**_**2**_ **(%)**	96 (85–98)	98 (87–98)
Qp (L/min)	4.31 (2.40–7.23)	5.00 (3.00–7.60)
Qs (L/min)	3.40 (2.60–4.30)	3.51 (2.80–4.67)
Qp:Qs	1.40 (0.69–2.17)	1.50 (0.72–2.20)
**Classification**		
Increased (Qp:Qs > 1.5)	514 (47.2)	194 (48.5)
Decreased (Qp:Qs < 0.9)	363 (33.4)	118 (29.5)
Normal (0.9 ≤ Qp:Qs ≤ 1.5)	211 (19.4)	88 (22.0)
Acyanotic CHD	634 (58.3)	239 (59.8)
**Shunt lesion**		
**Repaired**		
Atrial septal defect	382 (35.1)	180 (44.8)
Ventricular septal defect	129 (11.9)	38 (9.5)
PAPVC	11 (1.0)	7 (1.8)
Patent ductus arteriosus	34 (3.1)	2 (0.5)
Complete AV canal defect	6 (0.6)	2 (0.5)
AP window	4 (0.4)	0(0)
Coronary cameral fistula	3 (0.3)	0(0)
Palliated PAB	2 (0.2)	1 (0.2)
Unrepaired with PH	43 (4.0)	0 (0)
Valvular lesion	3 (0.3)	3 (0.8)
RVOTO/DCRV	16 (1.5)	6 (1.5)
ALCAPA	1 (0.1)	0 (0)
Cyanotic CHD	454 (41.7)	161 (40.2)
**Tetralogy of Fallot**		
Unrepaired	56 (5.1)	38 (9.5)
Repaired (with residual shunt)	8 (0.7)	0 (0)
Double outlet right ventricle (palliated BDGS)	30 (2.8)	5 (1.2)
**Dextro-transposition of great arteries**		
With VSD	10 (0.9)	3 (0.7)
With VSD/PS	11 (1.0)	0 (0)
Palliated BDGS	3 (0.3)	0 (0)
**Single ventricle**		
No PS	16 (1.5)	2 (0.5)
PS	23 (2.1)	7 (1.7)
**Palliated**		
PAB	7 (0.6)	7 (1.7)
BDGS	230 (21.1)	82 (20.5)
**Congenital corrected transposition**		
With LVOTO	8 (0.7)	0 (0)
**Palliated**		
BDGS	10 (0.9)	1 (0.3)
PAB	1 (0.1)	4 (1.0)
Other^a^	11 (1.0)	2 (0.5)
No major CHD	30 (2.8)	9 (2.3)

Values are presented as median (interquartile range) or number (%)

*ALCAPA* anomalous left coronary artery from pulmonary artery, *AP* aortopulmonary, *AV* atrioventricular, *BDGS* bidirectional Glenn shunt, *CHD* congenital heart disease, *DCRV* double-chambered right ventricle, *LVOTO* left ventricular outflow tract obstruction, *PAB* pulmonary artery banding, *PAPVC* partial anomalous pulmonary venous connection, *PH* pulmonary hypertension, *PS* pulmonic stenosis, *Qp* pulmonary blood flow, *Qp:Qs* pulmonary-to-systemic blood flow ratio, *Qs* systemic blood flow, *RVOTO* right ventricular outflow tract obstruction, *SpO*_*2*_, peripheral oxygen saturation measured by pulse oximetry, *VSD* ventricular septal defect

^a^Total anomalous pulmonary venous connection, truncus, criss cross hearts, and Fontan

### DLM-predicted and Fick-derived Qp:Qs

**Fig. 3:**
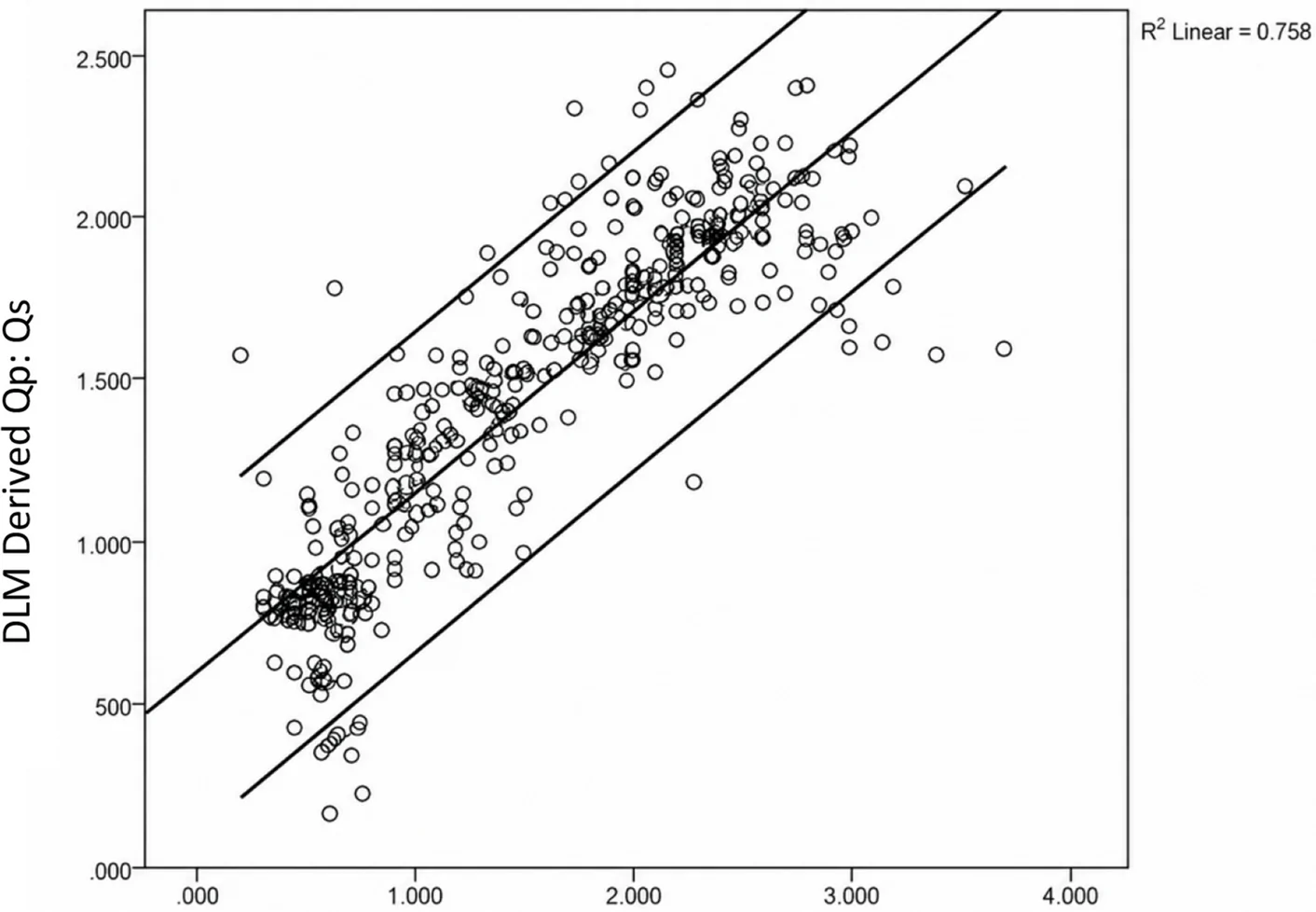
Scatter plot of deep learning method (DLM)-predicted pulmonary-to-systemic blood flow ratio (Qp:Qs) and Fick-derived Qp:Qs

**Fig. 4:**
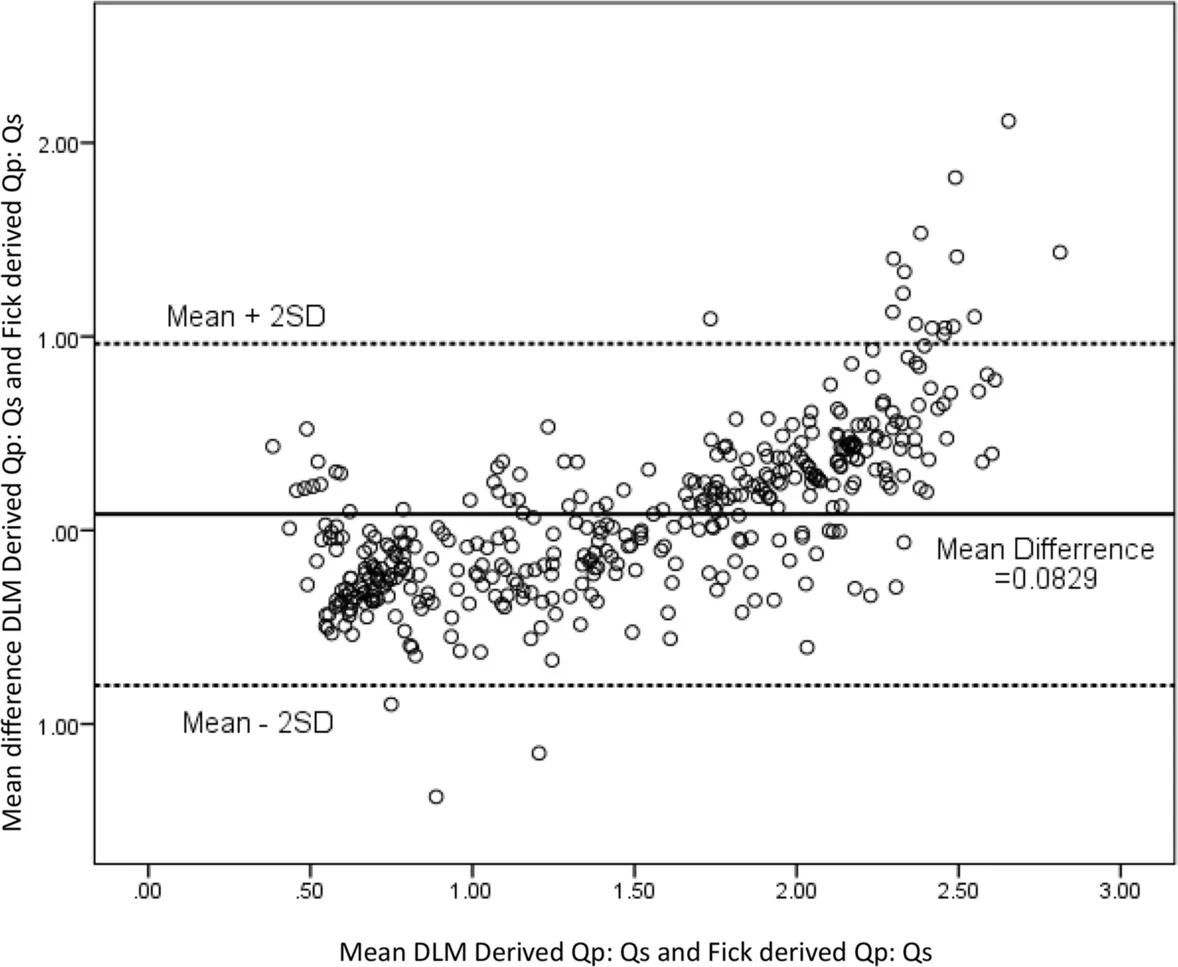
Bland–Altman plot comparing Fick-derived pulmonary-to-systemic blood flow ratio (Qp:Qs) and deep learning model (DLM)-predicted Qp:Qs. SD, standard deviation

The scatter plot and Bland–Altman plot comparing Fick-derived Qp:Qs and DLM-predicted Qp:Qs are depicted in Figs. 3 and 4. RMSE was 0.395, and intraclass correlation was 0.782, indicating that the DLM prediction is closer to actual Fick-derived values. Bland–Altman analysis demonstrated a bias value of 0.08, a precision of 0.44, an upper limit of agreement (mean + 2SD) of 0.96, and a lower limit of agreement (mean – 2SD) of –0.80. The analysis showed that the difference between DLM-derived Qp:Qs and Fick-derived Qp:Qs increases linearly with an increase in mean Qp:Qs until a ratio of approximately 2.5. Beyond a shunt ratio of 2.5, the DLM does not correlate as well with the Fick-derived Qp:Qs.

Interclass agreement between Fick-derived and DLM-predicted Qp:Qs classifications, assessed using Cohen κ, was 0.770 (*P* < 0.001), indicating substantial agreement.

**Fig. 5:**
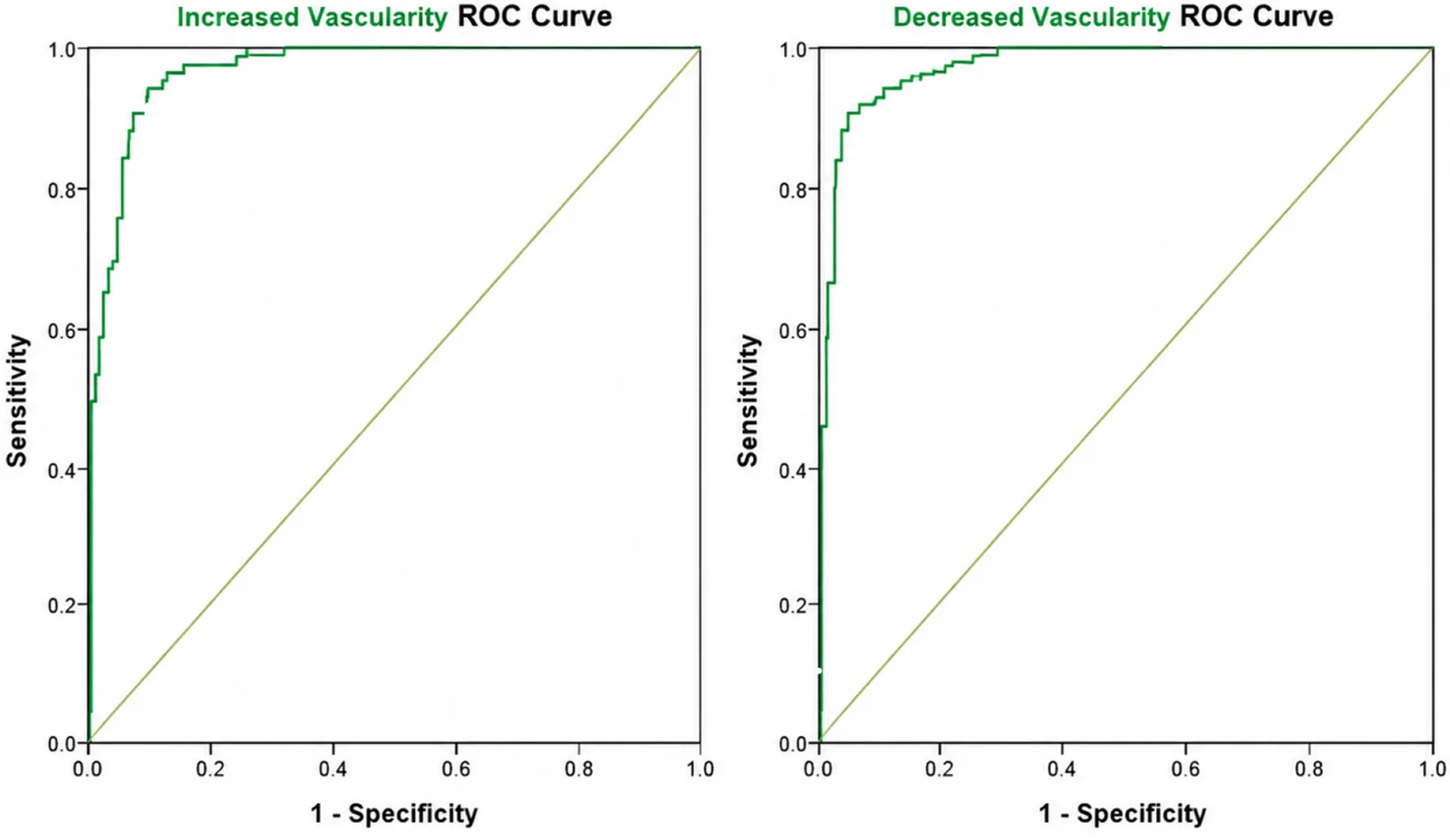
Receiver operating characteristic (ROC) curves for the deep learning model (DLM) predicting pulmonary-to-systemic blood flow ratio (Qp:Qs) > 1.5 and < 0.9. The green line represents the DLM-predicted Qp:Qs. The yellow line represents the reference line

For predicting a Qp:Qs of > 1.5, the DLM achieved a sensitivity of 98%, specificity of 90%, accuracy of 95%, and AUROC of 97%. For predicting QP:QS ratio of < 0.9, the model demonstrated a sensitivity of 92%, specificity of 95%, and AUROC of 98% (Fig. 5).

### Clinician interpretation and DLM prediction of pulmonary vascularity

Increased and decreased pulmonary vascularity were interpreted by experts and fellows with a concordance rate of 75% and 66%, respectively, compared to the DLM, which had a concordance rate of 88% and 98%, respectively (*P* = 0.03) (Table 3).

**Table 3:** The diagnostic concordance between the classification of pulmonary vascularity based on Fick-derived Qp:Qs and classification by clinicians

Classification by clinicians	Classification based on Fick-derived Qp:Qs		
	Increased (*n* = 194)	Decreased (*n* = 118)	Normal (*n* = 88)
Increased			
DLM (*n* = 216)	190 (88.0)	3 (1.4)	23 (10.6)
All clinicians (*n* = 157)	117 (74.5)	11 (7.0)	29 (18.5)
Decreased			
DLM (*n* = 92)	0 (0)	90 (97.8)	2 (2.2)
All clinicians (*n* = 83)	11 (13.3)	55 (66.3)	17 (20.5)
Normal			
DLM (*n* = 92)	4 (4.3)	25 (27.2)	63 (68.5)
All clinicians (*n* = 60)	31 (51.7)	9 (15.0)	20 (33.3)

Values are presented as number (%)

*DLM* deep learning model, *Qp:Qs* pulmonary-to-systemic blood flow ratio

### Interclinician agreement on pulmonary vascularity

Experts were found to have a moderate agreement (κ, 0.2–0.50; *P* < 0.001) with Fick-derived Qp:Qs classification, whereas fellows had a fair to moderate agreement (κ, 0.76–0.19; *P* < 0.001).

For interpreting increased pulmonary vascularity, experts had an average sensitivity of 61%, specificity of 74%, and accuracy of 68%, whereas fellows had an average sensitivity of 60%, specificity of 64%, and accuracy of 62% (Table 4).

**Table 4:** Sensitivity and specificity rates of individual experts and fellows for detecting increased vascularity

	Expert 1	Expert 2	Expert 3	Fellow 1	Fellow 2	Fellow 3
Sensitivity (%)	85.0	48.2	49.2	54.9	77.2	48.7
Specificity (%)	56.0	84.1	81.2	69.1	47.3	77.3
Positive predictive value	64.3	73.8	70.9	62.4	57.8	66.7
Negative predictive value	80.0	63.5	63.2	62.2	69.0	61.8
Accuracy (%)	70.0	66.8	65.8	62.3	61.8	63.5

The number of end-on vessels had a strong interobserver agreement, whereas individual peripheral vessels, size of end-on vessel, and branch pulmonary arteries with their respective bronchi and opacity of lung fields had modest agreement (all *P* < 0.001) (Table 5).

**Table 5:** Interobserver agreement of individual defining features of pulmonary vascularity on chest radiographs

Individual feature	Kendall W	*P*-value
1. RDPA and tracheal diameter	0.12	< 0.001
2. Size of adjacent bronchi and PA	0.35	< 0.001
3. Aortic knuckle and MPA	0.10	< 0.001
4. Individual peripheral vessels	0.41	< 0.001
5. No. of end-on vessels	0.60	< 0.001
6. End-on vessel and end-on bronchi	0.36	< 0.001
7. Bronchovascular markings	0.02	< 0.001
8. Opacity of lung fields	0.46	< 0.001
9. Heart size	0.20	< 0.001

*MPA* main pulmonary artery, *PA* pulmonary artery, *RDPA* right descending pulmonary artery

## Discussion

Our study evaluated pulmonary vascularity on CXRs by using a DLM and structured clinician assessment, and demonstrated that the DLM outperforms clinicians in both quantitative and objective interpretation.

We used transfer learning, which is the most effective way in designing a DLM, as demonstrated by previous studies interpreting CXRs and computed tomography [13–18]. Our ViT model performed better than the CNNs in terms of handling larger datasets, the self-attention mechanism, higher accuracy, and computational efficiency.

Augmentation methods such as random cropping, clipping, rotation, and CLAHE have been used previously to improve the performance of a DLM [26]. We used CLAHE and random flipping rotation in our study, but neither contributed to model performance. CLAHE may have distorted crucial features, while augmentation probably introduced unwanted variations. We obtained a low SD of the RMSE (± –0.03), indicating robust model performance and demonstrating excellent generalization capabilities.

The DLM-predicted and Fick-derived Qp:Qs had a significant correlation both quantitatively and categorically. The Bland–Altman analysis demonstrated linear agreement between the two methods up to a Qp:Qs of approximately 2.5. This could be because the training dataset contained fewer values above 2.5, hence explaining the possible reduction in the predictive ability of the DLM. Including more cases with Qp:Qs of > 2.5 may improve the predictive accuracy in this range.

In vascularity classification, DLM showed superior interpretation compared to clinicians with an accuracy rate of 95%. However, among the clinicians, experts performed better than the fellows.

Previous studies have evaluated individual features or a small group of features in interpreting pulmonary vascularity by clinicians [9, 11, 22]. In contrast, we included all the relevant features that predict pulmonary vascularity from CXRs. Additionally, we included a detailed definition of each feature to facilitate interpretation. The use of standardized, predefined criteria has enabled us to conclude that certain features on the CXR aid in predicting pulmonary vascularity more precisely than others, with an acceptable interobserver agreement.

Based on this research, we have developed an online application incorporating our DLM that allows users to upload a PNG radiograph and receive an output as a numerical value (Qp:Qs) [27]. Additionally, we have included a Grad-CAM visualization that shows how the model evaluates vascularity visually, emphasizing the key areas. This feature can assist the incorporation of AI into daily patient care for the interpretation of pulmonary vascularity. The platform could lead to more accurate classifications in the outpatient setting and increase the robustness of decisions about operability and medical therapy.

## Limitations

This is a single-center study, which may limit the generalizability of the findings. There are differences in radiographic acquisition protocols across centers and the possibility of the model learning institution-specific imaging characteristics. External validation using datasets from multiple centers is required before broader applicability can be established. Our model performance was not optimal when the Qp:Qs was more than 2.5. Expanding the training group dataset can potentially overcome this limitation. The AI-based model must also be tested in combination with other common clinical and noninvasive assessments of shunt ratios in real-world clinical settings to help fine-tune its application. Pulmonary vascular resistance was not incorporated into the prediction model and represents an important area for future study.

## Conclusions

DLM-based analysis of CXRs may enable a more precise quantitative evaluation of pulmonary vascularity, outperforming prespecified structured assessment by clinicians.

## Data Availability

No datasets were generated or analysed during the current study.

## Abbreviations

AI: Artificial intelligence
AUROC: Area under the receiver operating characteristic curve
CHD: Congenital heart diseases
CLAHE: Contrast-limited adaptive histogram equalization
CNN: Convolutional neural network
CXR: Chest radiograph
DLM: Deep learning model
IQR: Interquartile range
Qp:Qs: Pulmonary-to-systemic blood flow ratio
RMSE: Root mean squared error
SD: Standard deviation
timm: PyTorch Image Models
ViT: Vision Transformers

## References

[1] Swischuk LE, Stansberry SD. Pulmonary vascularity in pediatric heart disease. J Thorac Imaging. 1989;4:1–6. 2664205 10.1097/00005382-198907000-00003

[2] Viswanathan S, Kumar RK. Assessment of operability of congenital cardiac shunts with increased pulmonary vascular resistance. Catheter Cardiovasc Interv. 2008;71:665–70. 18360864 10.1002/ccd.21446

[3] Kumar S, Kumar RK. Common shunt lesions with pulmonary hypertension: who will benefit from surgery? Indian J Thorac Cardiovasc Surg. 2025;41:718–29. 40417595 10.1007/s12055-024-01786-7 PMC12102052

[4] Feltes TF, Bacha E, Beekman RH, Cheatham JP, Feinstein JA, Gomes AS, et al. Indications for cardiac catheterization and intervention in pediatric cardiac disease: a scientific statement from the American Heart Association. Circulation. 2011;123:2607–52. 21536996 10.1161/CIR.0b013e31821b1f10

[5] Marín Rodríguez C, Sánchez Alegre ML, Lancharro Zapata Á, Alarcón Rodríguez J. What radiologists need to know about the pulmonary-systemic flow ratio (Qp/Qs): what it is, how to calculate it, and what it is for. Radiol. 2015;57:369–79. 10.1016/j.rx.2015.04.001 26070521

[6] Hundley WG, Li HF, Lange RA, Pfeifer DP, Meshack BM, Willard JE, et al. Assessment of left-to-right intracardiac shunting by velocity-encoded, phase-difference magnetic resonance imaging. A comparison with oximetric and indicator dilution techniques. Circulation. 1995;91:2955–60. 7796506 10.1161/01.cir.91.12.2955

[7] Vonk-Noordegraaf A, van Wolferen SA, Marcus JT, Boonstra A, Postmus PE, Peeters JW, et al. Noninvasive assessment and monitoring of the pulmonary circulation. Eur Respir J. 2005;25:758–66. 15802353 10.1183/09031936.05.00122104

[8] Ravin CE. Pulmonary vascularity: radiographic considerations. J Thorac Imaging. 1988;3:1–13. 3336060 10.1097/00005382-198801000-00003

[9] Laya BF, Goske MJ, Morrison S, Reid JR, Swischuck L, Ey EH, et al. The accuracy of chest radiographs in the detection of congenital heart disease and in the diagnosis of specific congenital cardiac lesions. Pediatr Radiol. 2006;36:677–81. 16547698 10.1007/s00247-006-0133-2

[10] Birkebaek NH, Hansen LK, Elle B, Andersen PE, Friis M, Egeblad M, et al. Chest roentgenogram in the evaluation of heart defects in asymptomatic infants and children with a cardiac murmur: reproducibility and accuracy. Pediatrics. 1999;103:e15. 9925861 10.1542/peds.103.2.e15

[11] Tumkosit M, Yingyong N, Mahayosnond A, Choo KS, Goo HW. Accuracy of chest radiography for evaluating significantly abnormal pulmonary vascularity in children with congenital heart disease. Int J Cardiovasc Imaging. 2012;28(Suppl 1):69–75. 22628052 10.1007/s10554-012-0073-x

[12] Cardinale L, Priola AM, Moretti F, Volpicelli G. Effectiveness of chest radiography, lung ultrasound and thoracic computed tomography in the diagnosis of congestive heart failure. World J Radiol. 2014;6:230–7. 24976926 10.4329/wjr.v6.i6.230 PMC4072810

[13] Hua KL, Hsu CH, Hidayati SC, Cheng WH, Chen YJ. Computer-aided classification of lung nodules on computed tomography images via deep learning technique. Onco Targets Ther. 2015;8:2015–22. 26346558 10.2147/OTT.S80733 PMC4531007

[14] Ausawalaithong W, Thirach A, Marukatat S, Wilaiprasitporn T. Automatic lung cancer prediction from chest x-ray images using the deep learning approach. 2018 11th Biomedical Engineering International Conference (BMEiCON); 2018 Nov 21–24; Chiang Mai, Thailand. IEEE; 2018. p. 1–5.

[15] Lakhani P, Sundaram B. Deep learning at chest radiography: automated classification of pulmonary tuberculosis by using convolutional neural networks. Radiology. 2017;284:574–82. 28436741 10.1148/radiol.2017162326

[16] Rajpurkar P, Irvin J, Zhu K, Yang B, Mehta H, Duan T, et al. CheXNet: radiologist-level pneumonia detection on chest x-rays with deep learning [Preprint]. arXiv:1711.05225; [posted 2017 Nov 14]. 10.48550/arXiv.1711.05225.

[17] Kusunose K, Hirata Y, Tsuji T, Kotoku J, Sata M. Deep learning to predict elevated pulmonary artery pressure in patients with suspected pulmonary hypertension using standard chest x ray. Sci Rep. 2020;10:19311. 33203947 10.1038/s41598-020-76359-w PMC7672097

[18] Danilov VV, Makoveev AO, Proutski A, Ryndova I, Karpovsky A, Gankin Y. Explainable AI to identify radiographic features of pulmonary edema. Radiology Advances. 2024;1:umae003. 41059151 10.1093/radadv/umae003 PMC12429240

[19] Toba S, Mitani Y, Yodoya N, Ohashi H, Sawada H, Hayakawa H, et al. Prediction of pulmonary to systemic flow ratio in patients with congenital heart disease using deep learning-based analysis of chest radiographs. JAMA Cardiol. 2020;5:449–57. 31968049 10.1001/jamacardio.2019.5620 PMC6990846

[20] Arnois DC, Silverman FN, Turner ME. The radiographic evaluation of pulmonary vasculature in children with congenital cardiovascular disease. Radiology. 1959;72:689–98. 13658404 10.1148/72.5.689

[21] Yoo SJ, MacDonald C, Babyn P, editors. Chest radiographic interpretation in pediatric cardiac patients. Thieme; 2010.

[22] Coussement AM, Gooding CA. Objective radiographic assessment of pulmonary vascularity in children. Radiology. 1973;109:649–54. 4772181 10.1148/109.3.649

[23] Khalil M, Khalil A, Ngom A. A comprehensive study of vision transformers in image classification tasks [Preprint]. arXiv:2312.01232; [posted 2023 Dec 2]. 10.48550/arXiv.2312.01232.

[24] Wanto A, Yuhandri Y, Okfalisa O. Optimization accuracy of CNN model by utilizing CLAHE parameters in image classification problems. 2023 International Conference on Networking, Electrical Engineering, Computer Science, and Technology (IConNECT); 2023 Aug 25–26; Bandar Lampung, Indonesia. IEEE; 2023. p. 253–8.

[25] Hodson TO. Root-mean-square error (RMSE) or mean absolute error (MAE): when to use them or not. Geosci Model Dev. 2022;15:5481–7.

[26] Shorten C, Khoshgoftaar TM, Furht B. Text data augmentation for deep learning. J Big Data. 2021;8:101. 34306963 10.1186/s40537-021-00492-0 PMC8287113

[27] SuperSecureHuman. xray-reg. Hugging Face; [ 7 December 2025]. Available from: https://huggingface.co/spaces/SuperSecureHuman/xray-reg.

